# Nature and happiness in an individualist and a collectivist culture

**DOI:** 10.1038/s41598-022-11619-5

**Published:** 2022-05-11

**Authors:** Tal Svoray, Michael Dorman, Sarah Abu-Kaf, Golan Shahar, Robert Gifford

**Affiliations:** 1 Department of Geography and Environmental Development, Ben-Gurion University of the Negev, Beer-Sheva, Israel; 2 Department of Psychology, Ben-Gurion University of the Negev, Beer-Sheva, Israel; 3 Conflict Management & Resolution Program, Ben-Gurion University of the Negev, Beer-Sheva, Israel; 4 Department of Psychology, University of Victoria, Victoria, Canada

**Keywords:** Human behaviour, Environmental social sciences

## Abstract

According to the attention restoration theory, exposure to nature (ETN) renews one's capacity to focus attention, which decreases cognitive fatigue and therefore may increase positive emotions. Indeed, natural settings have been associated with high prevalence of happy facial expressions (HFE). However, how universal the association is, remains unclear. We explored the ETN-HFE association in Boston, US, representing a less collectivistic culture, and Yokohama, Japan, representing a more collectivistic one. Evidence from satellite images and social network data, using geoinformatics and statistical tools, revealed that individuals from both societies exhibited more happiness when they were photographed in more natural settings. These associations varied with temporal variations expressed through weekly and annual effects. In addition, we found that the presence of others was also associated with prevalence of HFE in natural settings at Yokohama and Boston but the relation was significantly stronger in Boston. Despite some relatively minor differences between the countries, these results support the universality of the association between ETN and HFE.

## Introduction

### Exposure to nature and happiness

Exposure to nature (ETN) is associated with increased well-being and happiness^1^, reduced stress^2^ and improved general life satisfaction^3^. Spending time in the wilderness can improve one's mental state and even green parks within the built environment may advance well-being^4^.

Individuals report less mental distress and greater well-being when they live in urban areas with more green space^5^ and occasional nature visits improve life satisfaction and well-being in general. Furthermore, nature, measured as a composite score based on distance from water bodies, green and underdeveloped built areas, has been significantly associated with happy facial expressions (HFE) of individuals, mainly during warm months^6^. Similarly, in the UK, MacKerron and Mourato^7^ found that research participants were significantly and substantially happier outdoors in green and other natural habitat types than they were in built environments.

One framework that predicts such a result is Attention Restoration Theory (ART)^8^, which suggests that nature―which is relatively undemanding for attentional resources―renews one’s cognitive capacity for focusing attention. In turn, individuals with renewed directed attention suffer less cognitive fatigue and experience positive emotions such as increased happiness.

### Collectivistic and individualistic dimensions

Individualism-collectivism has been identified as a central dimension of cultural diversity^9–11^. Collectivist cultures are characterized by special concern for and maintenance of relationships. People are interdependent within their group, especially the family, and they strive to promote what their group expects of them. In individualistic societies, people's main concern is their autonomy; They are independent of their inner groups, want to progress and to influence their social group^11,12^.

Kagitcibasi^13^ proposed a non-dichotomous model, according to which some con-temporary individualistic cultures also include the basic interdependence within the family that is characteristic of more collectivistic cultures. In other words, collectivism and individualism may be independent rather than dichotomous variables, and cultures may be similar in terms of one of these dimensions yet differ in regard to the other. A review article conducted by Ogihara^14^ pointed to an important trend of strengthening the dimension of individualism among Japanese society as a result of economic factors, strengthening of urbanization, divorce rate increased and household size decreased. As a result of this trend, more similarity in the individualism dimension is expected between the American society and the Japanese society. However, the same review article highlights the presence and importance of other social values associated with collectivism among Japanese society. Thus, we can assume that Japanese society still emphasizes more values of collectivism as compared to American society^14,15^.

### The role of cultural context in the ETN-HFE association

Facial expressions are a common form of nonverbal information transfer that may be used to convey emotional states of a person to other persons. A question exists about the universality of facial expressions^16,17^. Whereas Darwin^18^ claimed that facial expressions evolved to protect the human species from misinterpretation of the intentions of other individuals, others^19^ argue for a culture-dependent approach to facial expressions. In contrast, the effect of attention restoration should be universal, in that nature, regardless of place, is expected to provide renewal of the directed attention resource when it has been dissipated by everyday distractions, and therefore people should be happier in restorative natural sites. However, cross-national differences may not be necessarily equal, but ordered in the same way, because of the chronic and moment-to-moment salience of individualism and collectivism^20^.

For example, Japanese individuals, on average, apparently are more emotionally restrained than Americans, in both positive and negative emotions^21–23^. If the cultural effect on the ETN-HFE relation is indeed substantial, one would expect that individuals in Japan, a more collectivist culture, tend to upload fewer pictures with HFE in natural settings, compared to Americans, a less collectivistic culture. On the other hand, if the ETN-HFE effect is universal, Japanese individuals will also upload photos with HFE in natural sites and one would conclude that ETN activates the renewal of directed attention in individuals in both groups, despite the cultural differences.

As for the effect of others, Japanese lay theories conceive of emotions as interpersonal, because of their tendency to regard most behavior as relational^24^. In contrast, American lay theories conceive of emotions as intrapersonal because of that culture’s tendency to regard most behavior as independent of others. Consequently, in Japanese contexts, emotions are more likely to be expressed when relationships are salient. Thus, compared to Americans, Japanese individuals should be more emotional or happier in social contexts and less happy outside such contexts, for example when they are not with others^25,26^.

### Aim and objectives

The aim of this study was to determine whether exposure to nature is associated with happy facial expressions similarly in individuals from collectivist (Yokohama, Japan) and individualist (Boston, MA) societies. It had three specific goals: (1) to investigate whether the ETN-HFE link is universal, that is, whether the associations are the same in Boston and Yokohama; (2) to assess the effect on HFE of other spatio-temporal trends—diurnal, monthly, and seasonal—and whether those effects vary among regions; and (3) to investigate whether the presence of other people affects HFE, including whether that differs in individualist and collectivist societies.

## Methods

### Data retrieval from Flickr

**Figure 1:**
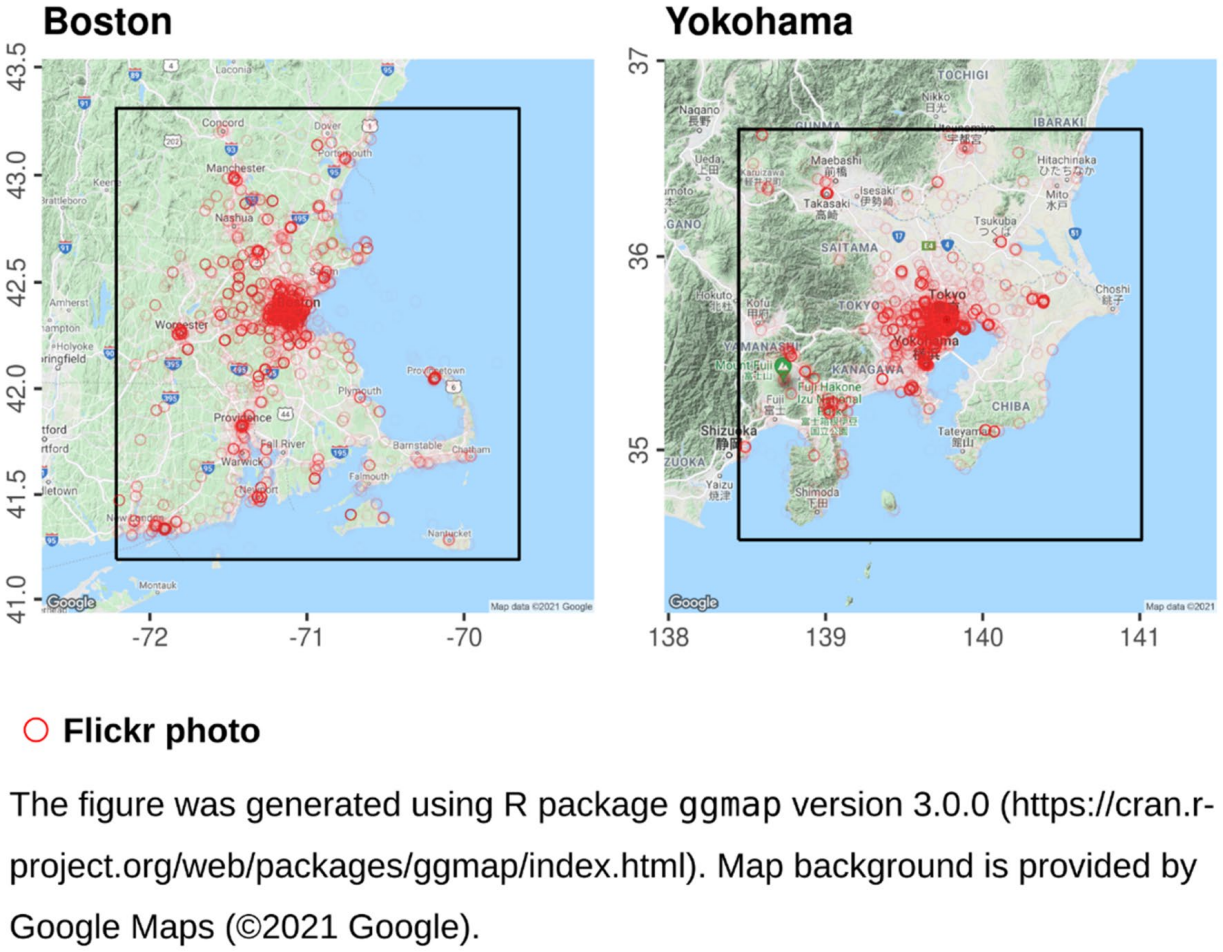
The Boston and Yokohama study areas. Circles mark the locations of analyzed Flickr photos that include human faces (N = 62,642 in Boston; N = 74,619 in Yokohama).

Data for the Greater Boston Area (GBA) and Yokohama regions were downloaded from Flickr, the image hosting service. Those areas were selected because Flickr usage is particularly high in these cities and because of their environmental and demographic similarity. Both are large port cities (Fig. 1) and they have similar land area size (26,021 and 32,256 km^2^, in Boston and Yokohama, respectively). However, the population of Yokohama (43.6 × 10^6^) is much greater than that of Boston (8.4 × 10^6^) while population estimates are based on the “Gridded Population of the World (GPW), v3” dataset for 2015^27^. All 1,018,349 and 1,562,266 public Flickr photos taken in Boston and in Yokohama over four years (2012–2015) were downloaded, along with their metadata, using the Flickr API (https://www.flickr.com/services/api/). Flickr images are associated with a label describing the recorded accuracy of location information, ranging from the lowest—world (1), through country (3), region (6) and city (11), and up to the most proximate category, street-level (https://www.flickr.com/services/api/flickr.photos.search.html) (16). Only images associated with the most proximate geo-tag (“street-level”) were retained, resulting in 525,689 and 1,350,768 images in Boston and Yokohama, respectively. The number of geo-tagged photos, per capita, over the course of 4 years, was therefore twice as high in Boston (0.06) as in Yokohama (0.03). Only photos including faces were retained. This resulted in the final sample size of 62,642 photos in Boston and 74,619 photos in Yokohama (Fig. 1).

Microsoft’s Emotion API^28^ (https://www.microsoft.com/cognitive-services/en-us/emotion-api) was used to recognize the most-likely facial expressions of each imaged person in each Flickr photo. Emotion API produces a likelihood score (between 0 and 1) for each of eight emotions: anger, contempt, disgust, fear, happiness, neutral, sadness, and surprise. The number of people photographed in each photo was also recorded. Following API documentation, the most likely emotion for each face was selected, based on score maxima. The proportion of “happy” faces out of the total number of faces, per photo, comprised the dependent variable.

The proportion and sample size of happy expressions among the sampled photos, per study area and studied environmental variables, are given in Supplementary Fig. S1 in the Supplementary Materials.

The Emotion API has been utilized efficaciously in studies of emotion recognition^6,29,30^. For example, the Emotion API and the analogous Vision API (by Google) were recently used to detect sentiment in Flickr photos to investigate the spatial pattern of sentimental responses to stock market value^31^ and to natural disaster events^32^.

### Spatial predictors and ETN score

Three measures were used to represent ETN in the two study sites: green vegetation cover, proximity to water bodies, and proportion of undeveloped area. First, green vegetation cover was assessed using the Normalized Difference Vegetation Index (NDVI)^33^. Landsat-8 satellite images, which have a resolution of 30 m, were used to calculate NDVI images. The Landsat images were accessed from the Google Earth Engine platform^34^, based on the USGS Landsat-8 Collection-1 Tier-1 TOA Reflectance^35^ collection.

Complete images covering the two study areas were constructed by mosaicking and averaging all satellite images taken during a three-month period that coincided with the study period (2014-06-01 to 2014-08-31). We assumed that green vegetation did not change substantially during the period and, consequently, that the three months were representative of the entire study period (i.e., 2012–2015). A total of 36 and 25 of these Landsat images were used for the Boston and Yokohama areas, respectively. The ee.Algorithms.Landsat.simpleCloudScore function was used to filter out clouded pixels, which result in unreliable NDVI scores, before mosaicking. Finally, the NDVI layer was filtered with a 3 × 3 pixel focal window and the "average" function, so that the value associated with any given Flickr image expressed average green vegetation cover in the proximate neighborhood of 90 × 90 m.

Second, distance from water bodies was determined using the SRTM Water Body Dataset (https://lta.cr.usgs.gov/srtm_water_body_dataset), provided by the USGS. Although the Water Body Dataset is a vector layer (i.e., polygons), it has the same accuracy as the NDVI layer, because it is based on SRTM data with 30 × 30 m resolution.

Third, proportion of underdeveloped area was determined based on the 0.5 × 0.5 km MODIS-based Global Land Cover Climatology dataset^36^. This dataset is comprised of a 17-class land cover classification scheme, based on MODIS satellite observations during the period 2001–2010. To express urban development, we re-classified the land cover product into two categories: developed = 0 and undeveloped = 1, based on whether the land cover class in the photo was “Urban and Built-Up” (0) or any of the other 16 categories. The proportion of undeveloped area in a 1500 × 1500-m area was then calculated via filtering using a-3 × 3 pixel focal window.

An ETN composite index was computed by scaling each of the three naturalness components (NDVI, negative distance from water, and undeveloped area proportion) to a mean of 0 and standard deviation of 1. Note that naturalness denotes hereafter natural setting^37^ and refers to restorative open areas in the wilderness out of Boston, as well as to parks and undeveloped areas within the city. Then we computed their average per observation, reflecting the composite naturalness score for that particular observation while giving equal weight to each of the three components. It is important to emphasize that the variables were scaled using the mean and standard deviation of the data from both study areas combined. That way, absolute differences in naturalness between areas, such as lower vegetation cover in Yokohama (Table 1) are not masked by the standardization process. Creating a single ETN variable made evaluating its composite effect while factoring out the effect of temporal interactions possible. Including all three naturalness components and their interactions with study area would have resulted in a model with six separate coefficients for the naturalness effects and their differences across study areas, which would make it difficult to interpret and evaluate the overall effect of ETN. Instead, we fitted a composite model to evaluate the overall ETN effect, and three separate models to evaluate the effect of each ETN component (see below). The reason for using the negative distance from water is that we assume that naturalness increases with proximity to water. Thus, the three variables are consistent (higher value = more naturalness).

**Table 1:** Means and standard deviations of the dependent variable (HFE) and the independent variables in Boston and Yokohama. Note that the "Composite ETN", "Green", "Undeveloped" and "Near water" variables were scaled to mean 0 and standard deviation of 1 according to their distribution in both study areas *combined*. The fact that the means of those four variables were positive in Boston and negative in Yokohama therefore means that Yokohama was characterized by relatively lower naturalness compared to Boston.

Variable	Boston	Yokohama
HFE	0.51 (0.48)	0.46 (0.48)
Number of people	1.38 (0.77)	1.30 (0.70)
Composite ETN	0.37 (0.52)	− 0.31 (0.66)
Green	0.40 (1.06)	− 0.34 (0.80)
Undeveloped	0.19 (1.01)	− 0.16 (0.96)
Near water	0.50 (0.39)	− 0.42 (1.15)
Daytime	0.70 (0.46)	0.62 (0.48)
Weekend	0.49 (0.50)	0.46 (0.50)
Warm months	0.61 (0.49)	0.52 (0.50)

### Temporal predictors

Photo timestamps were classified according to three temporal cycles: diurnal, weekly, and seasonal. In terms of the diurnal cycle, photos were classified as “night” or “day,” according to the sunrise and sunset times. The latter were calculated separately for each photo, based on the spatial location and timestamp of each Flickr photo, using NOAA’s astronomical algorithm implemented in R 3.5.0^38^ package maptools^39^. The weekly cycle variable was calculated as the day of the week when the photo was taken, as identified from the photo metadata, and then used to classify photos into “work-week” (Monday–Friday) and “weekend” (Saturday–Sunday). In terms of the seasonal cycle, timestamps were classified as “cold” (Nov–Apr) or “warm” (May–Oct) seasons of the year.

### Statistical procedure

The link between ETN and temporal cycles on the prevalence of HFE was tested using binomial Generalized Linear Mixed Models (GLMM), also known as mixed-effects logistic regression models, and fitted using Penalized Quasi-Likelihood with R package MASS^40^. The outcome variable in all analyses was the proportion of happy faces (HFE) per photo including the number of faces with and without HFE, i.e., the number of “successes” and “failures” in binomial GLMM terminology. For each photo, the set of descriptive measures was based on its point location and instance timestamp. For example, the proportion of undeveloped area was based on the value in the particular grid cell where the point fell, which represents the proportion in a 1500 × 1500 m neighborhood (see above). The distance from water bodies was determined based on the shortest geometric distance between the point and the nearest shoreline, and so on.

In spatial data, when proximate points are more similar than distant ones, the actual sample size is smaller than the number of observations. This can lead to overestimated significance because “In autocorrelated data, each measurement does not contribute a full degree of freedom to the analysis, so degrees of freedom in statistical tests are over‐estimated, and this inflates the Type‐I error rate (falsely rejecting true null hypotheses.)”^41^. To avoid this source of error, a spatial autocorrelation structure among images taken on the same date was incorporated in all models^6^. Specifically, the model used included a spatial correlation term of exponential form, expressing the correlation between spatially proximate images taken on the same day.

For example, the R expression for fitting the first “baseline” model (Table 2) is shown below. The glmmPQL function (from package MASS) fits a Generalized Linear Mixed Model, where the fixed parameter specifies the list of dependent and independent fixed variables (see below). The correlation parameter specified the spatial correlation structure, in this study, based on the geographical distance between images taken on the same day in the same area, and the random parameter specifies the random effect of different days in different areas.

**Table 2:** The ETN composite association with HFE, its standard error, the test statistic (*t*-value), the *p*-value, the odds ratio and its 95% confidence interval.

Term	Estimate (slope)	Standard error	*t*	*p*	Odds ratio	Odds ratio ci
(Intercept)	− 0.73	0.03	− 21.71	< 0.001	0.48	[0.45, 0.51]
Yokohama	0.53	0.04	11.93	< 0.001	1.71	[1.56, 1.86]
Composite ETN	0.28	0.02	16.81	< 0.001	1.32	[1.28, 1.37]
Number of people	0.40	0.01	46.51	< 0.001	1.48	[1.46, 1.51]
Daytime	− 0.03	0.02	− 1.38	0.169	0.97	[0.94, 1.01]
Weekend	0.07	0.04	2.03	0.043	1.08	[1.00, 1.16]
Warm months	0.18	0.03	5.26	< 0.001	1.20	[1.12, 1.28]
Yokohama: composite ETN	− 0.09	0.02	− 4.19	< 0.001	0.92	[0.88, 0.95]
Yokohama: number of people	− 0.27	0.01	− 24.86	< 0.001	0.77	[0.75, 0.78]
Yokohama: daytime	0.04	0.03	1.61	0.108	1.04	[0.99, 1.09]
Yokohama: weekend	− 0.12	0.05	− 2.42	0.016	0.89	[0.81, 0.98]
Yokohama: warm month	− 0.21	0.05	− 4.51	< 0.001	0.81	[0.74, 0.89]

fit = glmmPQL(

cbind(happy, non_happy) ~

area + composite + n_people +

daytime + weekend + warm +

area:composite +

area:n_people +

area:daytime + area:weekend + area:warm,

data = dat,

family = binomial,

correlation = corExp(form = ~ x_utm + y_utm | area_date),

random = ~ 1 | area_date

)

The data file for reproducing this model, and the other ones (Tables 3, 4, 5), is available on Google Drive (see Data availability statement below).

**Table 3:** The association of less built-up areas association with HFE.

Term	Estimate (slope)	Standard error	*t*	*p*	Odds ratio	Odds ratio CI
(Intercept)	− 0.66	0.03	− 19.74	< 0.001	0.52	[0.48, 0.55]
Yokohama	0.44	0.04	9.98	< 0.001	1.56	[1.43, 1.70]
Undeveloped	0.18	0.01	20.85	< 0.001	1.2	[1.18, 1.22]
Number of people	0.39	0.01	46.36	< 0.001	1.48	[1.46, 1.51]
Daytime	− 0.02	0.02	− 0.95	0.343	0.98	[0.95, 1.02]
Weekday vs. weekend	0.08	0.04	2.19	0.029	1.08	[1.01, 1.16]
Warm months	0.18	0.03	5.08	< 0.001	1.19	[1.11, 1.28]
Yokohama: underdeveloped	− 0.01	0.01	− 0.87	0.383	0.99	[0.97, 1.01]
Yokohama: number of people	− 0.26	0.01	− 24.82	< 0.001	0.77	[0.75, 0.78]
Yokohama: daytime	0.02	0.03	0.99	0.32	1.03	[0.98, 1.08]
Yokohama: weekend	− 0.12	0.05	− 2.5	0.012	0.88	[0.80, 0.97]
Yokohama: warm months	− 0.21	0.05	− 4.48	< 0.001	0.81	[0.74, 0.89]

**Table 4:** The association of distance from water bodies with HFE.

Term	Estimate (slope)	Standard error	*t*	*p*	Odds ratio	Odds ratio CI
(Intercept)	−0.80	0.04	− 22.7	< 0.001	0.45	[0.42, 0.48]
Yokohama	0.54	0.05	11.67	< 0.001	1.71	[1.56, 1.87]
Near water	0.25	0.02	11.73	< 0.001	1.29	[1.23, 1.34]
Number of people	0.38	0.01	45.39	< 0.001	1.47	[1.44, 1.49]
Daytime	0.00	0.02	0.12	0.905	1	[0.97, 1.04]
Weekend	0.10	0.04	2.67	0.008	1.1	[1.03, 1.18]
Warm months	0.21	0.03	5.91	< 0.001	1.23	[1.15, 1.32]
Yokohama: near water	− 0.22	0.02	− 9.7	< 0.001	0.8	[0.77, 0.84]
Yokohama: number of people	− 0.26	0.01	− 24.32	< 0.001	0.77	[0.76, 0.79]
Yokohama: daytime	0.05	0.02	2.07	0.038	1.05	[1.00, 1.11]
Yokohama: weekend	− 0.14	0.05	− 2.75	0.006	0.87	[0.79, 0.96]
Yokohama: warm months	− 0.24	0.05	− 5.07	< 0.001	0.79	[0.72, 0.86]

**Table 5:** The association of green vegetation with HFE.

Term	Estimate (slope)	Standard error	*t*	*p*	Odds ratio	Odds ratio CI
(Intercept)	− 0.68	0.03	− 20.06	< 0.001	0.51	[0.48, 0.54]
Yokohama	0.42	0.04	9.45	< 0.001	1.53	[1.40, 1.67]
Green	0.01	0.01	0.84	0.403	1.01	[0.99, 1.02]
Number of people	0.39	0.01	45.86	< 0.001	1.48	[1.45, 1.50]
Daytime	− 0.01	0.02	− 0.46	0.645	0.99	[0.96, 1.03]
Weekend	0.09	0.04	2.45	0.014	1.09	[1.02, 1.18]
Warm months	0.21	0.04	6.07	< 0.001	1.24	[1.15, 1.32]
Yokohama: green	0.06	0.01	4.65	< 0.001	1.06	[1.04, 1.09]
Yokohama: number of people	− 0.26	0.01	− 24.53	< 0.001	0.77	[0.75, 0.79]
Yokohama: daytime	0.05	0.03	2.01	0.044	1.05	[1.00, 1.10]
Yokohama: weekend	− 0.14	0.05	− 2.72	0.007	0.87	[0.79, 0.96]
Yokohama: warm months	− 0.24	0.05	− 5.13	< 0.001	0.78	[0.71, 0.86]

The baseline statistical model included facial expression (proportional, count of “Happy” and “Neutral” expressions in the photo) as the dependent variable, in relation to the following independent variables:

1. Study area (categorical, “Boston” or “Yokohama”)
2. Composite ETN effect (the average of the three standardized components)
3. Number of people in the photo (count, whole positive numbers)
4. Daytime (categorical, “day” or “night”)
5. Weekend (categorical, “working-week” or “weekend”)
6. Warm months (categorical, “warm” or “cold”)
7. Five two-way interactions: area × composite, area × number_of_people, area × daytime, area × weekend, and area × warm months

To examine the effect of separate ETN components, rather than merely the ETN composite index, the above model was re-fitted three more times while replacing the composite ETN effect with each of its three components: less built-up areas, greater proximity to water, and more greenness.

Subsequently, the effects of composite ETN, its three separate components, and the three temporal cycle effects were examined. A slope analysis was undertaken only on effects for which the interaction with study areas was significant, implying that the effect in question differed in the two study areas. Specifically, the models were re-fitted while reversing the role of the reference level (i.e., dummy variable of 0) between the two study areas, Boston and Yokohama, so that the estimates for each of the study area could be reasonably compared (Table 6).

**Table 6:** The relation of ETN and temporal cycles phase with HFE prevalence.

Term	Boston				Yokohama			
	Estimate (slope)	Standard error	*t*	*p*	Estimate (slope)	Standard error	*t*	*p*
Composite ETN	0.28	0.02	16.81	< 0.001	0.19	0.01	15.14	< 0.001
Near water	0.25	0.02	11.73	< 0.001	0.03	0.01	4.77	< 0.001
Green	0.01	0.01	0.84	0.403	0.07	0.01	6.66	< 0.001
Num. of people	0.40	0.01	46.51	< 0.001	0.13	0.01	20.09	< 0.001
Weekend	0.07	0.04	2.03	0.043	− 0.05	0.03	− 1.36	0.173
Warm months	0.18	0.03	5.26	< 0.001	− 0.03	0.03	− 0.91	0.363

Standardized coefficient estimates are shown for those terms where the interaction with “study area” was significant, i.e., the relations differed among areas.

The estimates of ETN and the temporal effects were obtained from the baseline statistical model. The estimates of the three separate ETN components effects were obtained from the re-fitted models, where the effect of ETN was replaced with the effect of one of the three components of ETN.

Confidence intervals for predicted values were calculated using R package AICcmodavg^42^ (Fig. 3). Confidence intervals of the estimates were calculated using package nlme^43^ (Supplementary Table S1). Odds ratios and their confidence intervals were calculated by taking the exponent of the estimates and the estimate confidence intervals, respectively.

### Research ethics statement

The present study used only publically available Flickr images, accessible through the Flickr API service. The information we used included only the photo location (longitude/latitude) and capture timestamp (date + time). Image facial expressions were estimated using the Microsoft’s Emotion API, by passing the public image URL to the Emotion API service; no local copies of the images were made at any time. The Ethics Committee of the Department of Psychology at Ben-Gurion University of the Negev approved this research.

## Results

Descriptive statistics for the variables in Boston and Yokohama are presented in Table 1. The average number of people in a photo was very slightly higher in Boston—1.38 (*SD* = 0.77), compared to 1.30 (*SD* = 0.70) in Yokohama. The average composite ETN score was substantially higher in Boston − 0.37 (*SD* = 0.52), compared to − 0.31 (SD = 0.66) in Yokohama. All other variables (HFE, undeveloped areas, near water, green, daytime, weekend and warm season) were also higher in Boston.

**Figure 2:**
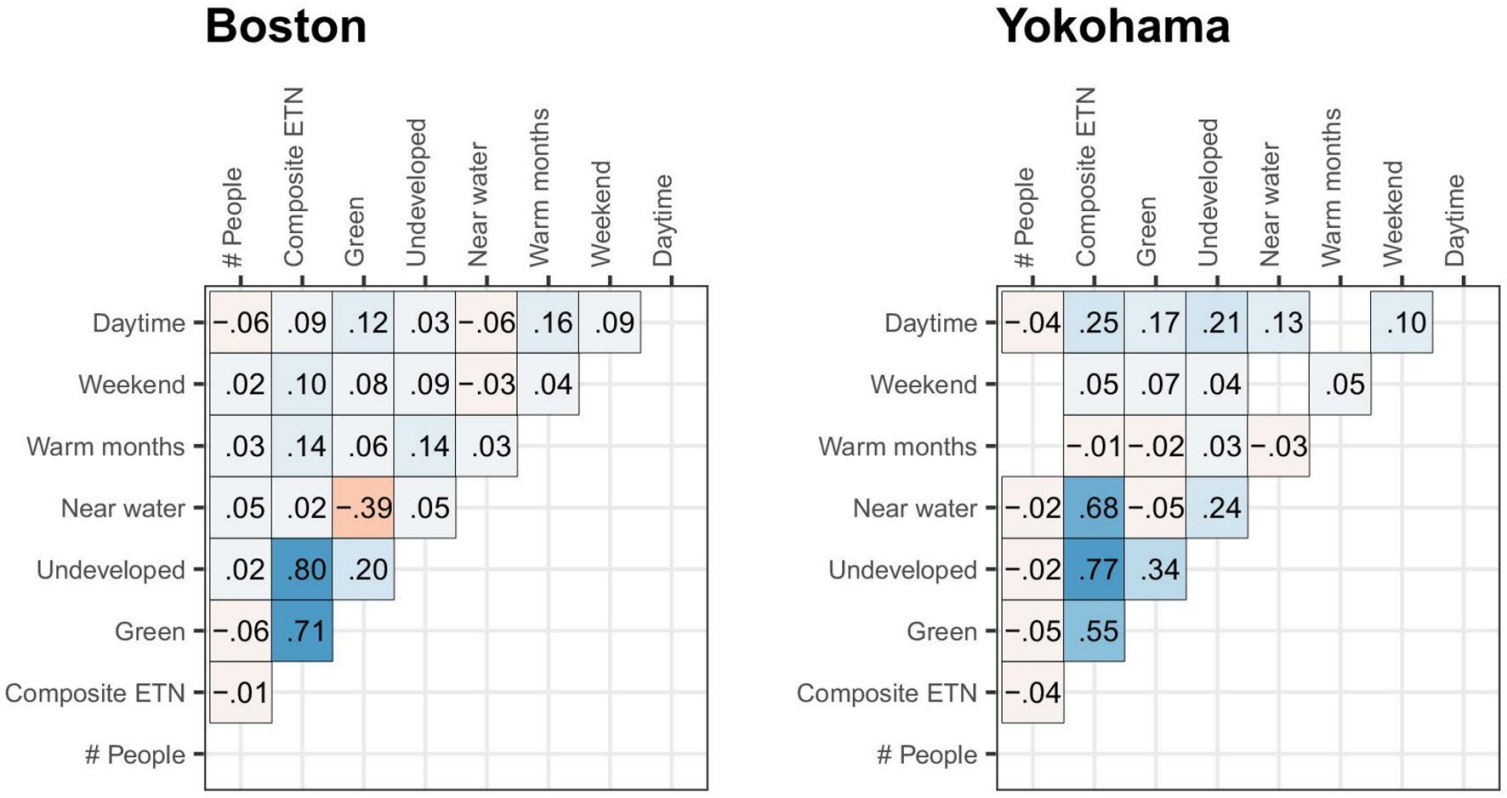
Pearson correlation values between explanatory variables, separately for each of the two studied regions. Non-significant (*p* > 0.05) coefficients are not shown.

Pairwise correlations between the variables, for each study area, are shown in Fig. 2. Strong positive correlations (*r* ≥ 0.5) were observed between the composite ETN score and each of its three components: underdeveloped areas, distance from water and greenness (except for proximity to water in Boston, which was weakly correlated with ETN, *r* = 0.02). All other correlations were low (*r* ≤ 0.4) or nonsignificant. This supports the validity of including the ETN score, or one of its components, in the same model along with the other environmental descriptors (Tables 2, 3, 4, 5).

**Figure 3:**
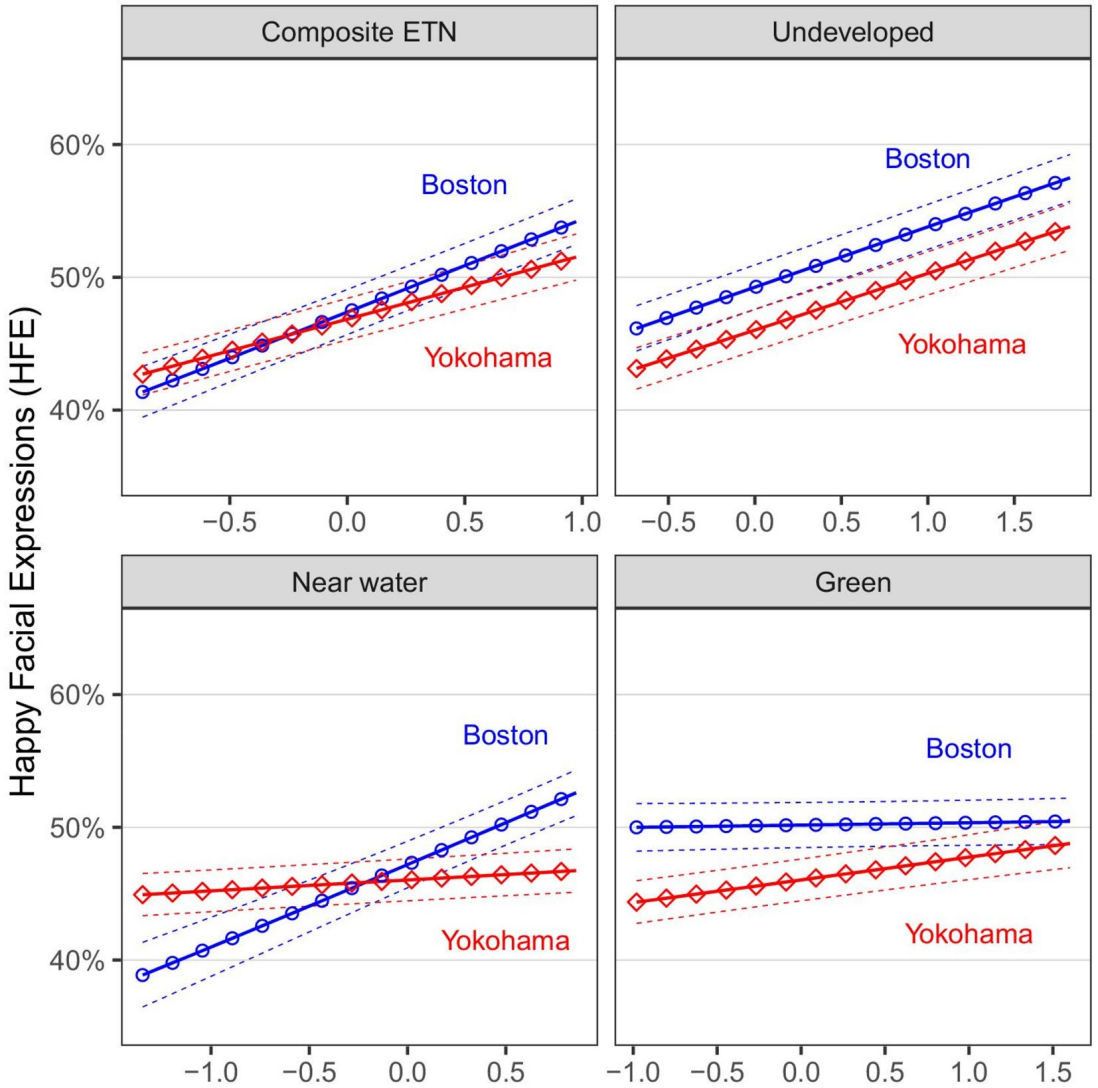
Predicted proportion of happy facial expressions (HFE) in a Flickr photo, as a function of composite ETN and its components, in Boston and Yokohama. All independent variables except for HFE were held fixed at 1. Higher values on the x-axis reflect more naturalness, less developed areas, greater proximity to water bodies and greater green vegetation cover. The values on the x-axis reflect the range between the 10% and 90% quantiles in the observed data. The solid lines represent predicted values, and the dashed lines represent 95% confidence intervals.

The ETN composite model and the three separate ETN component model associations with HFE are presented in Tables 2, 3, 4, 5. The association of ETN, its components and the number of people in the photo on HFE were significantly positive in both study areas, with the exception of green vegetation cover which was nonsignificant in Boston (Fig. 3, Table 6). This suggests that HFEs were more likely in natural, less built-up, proximate-to-water, and green areas, and when more individuals were photographed together. The diurnal relation with HFE was not significant, while the relation of warm months was significantly positive only for Boston (Table 6). Significant interactions with study area, reflecting the varying associations between Boston vs. Yokohama, were explored using slope analysis, as follows (Table 6).

Significant interactions with study area were observed for six of eight variables, implying that their association with HFE differed between Boston and Yokohama: composite ETN, distance from water, greenness, number of people in the photo, weekend, and warm months (Table 6). The composite ETN and proximity to water association with HFE were positive in both areas, yet significantly higher in Boston (0.28 and 0.25, respectively) compared to Yokohama (0.19 and 0.03) (Table 6, Fig. 3).

In contrast, the association of green vegetation cover with HFE was nonsignificant in Boston but significantly positive (0.07) in Yokohama (Table 6, Fig. 3). The number of people photographed associated with the prevalence of HFE was positive in both areas, but significantly weaker in Yokohama (0.13) than in Boston (0.40). The association of weekends and the warm months with HFE was positive in Boston (0.07 and 0.18, respectively), but nonsignificant in Yokohama (Table 6).

To evaluate correlations, the difference between predicted HFE (%) when the examined independent variable increased from its observed 10% quantile to 90% quantile was calculated, while all other independent variables were kept constant, i.e., the y-axis extent for each of the eight lines shown in Fig. 3^44^. The predicted effect magnitude of ETN and its components on HFE ranged between 0 and 14% (all effects were positive) (Fig. 3). For example, the strongest estimated effect was that of proximity to water in Boston, where predicted proportion of HFE increased by 14%, from 39% in areas where proximity to water was as low as the 10% quantile, to 53% in areas where proximity to water was as high as the 90% quantile (Fig. 3).

## Discussion

Very little research has examined whether exposure to nature is associated with happy facial expressions in both individual and collectivist societies. Previous research on this question is rather limited. Especially lacking are studies that used large databases extracted from social network data from specifically-chosen geographic locations. Using 4 years’ worth of data, we showed that individuals from both societies exhibited more happiness when they were photographed in more natural settings. These associations varied with temporal variations expressed through weekly and annual effects. In addition, we found that the presence of others was also associated with prevalence of HFE in natural settings at Yokohama and Boston but the relation was significantly stronger in Boston (Table 2). Our previous study^6^ explored the effect of environmental settings on happy facial expression, however, was limited to an American society and did not examine the effect of others. Such a study is limited by cultural effects and cannot provide evidence whether the beneficial effect of nature is limited to a specific society. The current study elaborates on this subject.

### Universal vs. culture-based outcomes

The question of ubiquity has long been pursued by psychologists, to better understand the effect of culture on human functioning. The association of culture, societal behavior, education and symbols with perception, cognition and emotions may be present but the question is whether they are substantial and, if the answer is yes, are they strong enough to mask the main effect of ETN on HFE, for example. Within this context, the association of individualistic vs. collectivistic societal differences with HFE is among the most significant questions about human behavior^11,20^. Large variations occur in the responses to external stimuli of individuals from the two cultural contexts^45,46^.

Evidence about the response of individuals from the two societies to ETN will allow better understanding of the association between nature and humans and the consequence of the involvement of others in this association. We examined whether these cultural differences would predict differences in response to ETN through facial expressions. An earlier study showed that proportions of pictures with HFEs are larger when people are present in places exposed to nature than in other places in a less collectivistic society, such as Boston^6^.

The present results show that individuals upload or share more photos with HFE when they are in natural areas compared with other parts of Yokohama as well. Significantly greater proportions of HFE occurred in the natural and less built-up areas in Boston and in Yokohama. These results are consistent with the assumption that the association between ETN and HFE is universal.

A question may arise as to whether people are happier in nature or tend to upload more happy photos when they visit nature. The present study did not explore this question and we are not aware of any evidence on the subject, but according to the plethora of studies of the association between ETN and emotions, we assume that ETN leads to increased positive emotions (see in the “Introduction” chapter). ETN, assessed as a composite measure of water bodies-green areas-low density built areas, yielded larger proportions of happy-face photos in both Boston and Yokohama, over a period of four years. We also assume that effects such as the personality of the photographed persons or instructions by the photographer to smile occur equally in different land uses and are not biased to any specific land-use.

These results suggest that ETN, and the effects of green areas, low-density built areas and proximity to water in particular, increase happiness in humans regardless of culture. That is despite the fact that previous studies have shown that in the Japanese context, individuals’ level of expression of happiness is often restrained in public^23,25,47–50^. The results show that ETN is positively associated with happiness despite differences in emotional display in these two cultural contexts. This supports previous observations about the effect of nature on human emotions and indicates that nature may be beneficial whether one is part of a group that emphasizes close relationships or one that emphasizes personal achievements.

However, this near-universal pattern is qualified to some extent by the analyses of the three ETN components separately, which revealed differences between the associations of distance from water and greenness with HFE in the two study sites. Distance from water had a statistically significant positive relation with HFE in both Boston and Yokohama, but the effect size statistically significantly differed: the positive relation of proximity to water with HFE was significantly stronger in Boston than in Yokohama (Tables 4, 6). Greenness had a significant positive relation with HFE in Yokohama, but no significant relation was found in Boston (Tables 5, 6).

The difference in the effect of proximity to water in Yokohama may have occurred because very few places in Yokohama exist where the coastline is open to the general public (i.e., most is occupied by industrial facilities or port infrastructure). Thus, in Yokohama, this potential nature resource is less accessible for public use, and therefore may be valued less because of fewer visits. In contrast, greater Boston covers an area of 49,815.28 km^2^, which includes 26,021.38 km^2^ of land (52%) and 23,763.7 km^2^ of water (48%) and therefore the city dwellers may have greatly accessibility to bodies of water and may enjoy the restorative services they provide more.

In addition, the population density of Japanese cities is relatively high and can be related to the geographical characteristics of Japan, which is characterized by large forest and mountainous areas, with few dwellings in the mountainous areas because of the topographic limitations. The population density in Yokohama (8534/km^2^) is high, about 60 percent greater than that of Boston (5344/km^2^).

It should be noted that computer vision face detection algorithms may exhibit gender and racial biases, due to, e.g., biased representation of age groups and races in the training datasets of those algorithms^51,52^. We could not locate specific studies on bias in emotion classification among American and Japanese societies, or racial groups in general^53^. Therefore, we cannot rule out bias in HFE detection among the two studied societies. However, we assume that any such bias is uniform within each of the study areas, therefore "factored out" by the study-area main effect in the models, and thus the estimates of spatial and temporal effects (which the study focused on) are expected to be unbiased.

### Temporal interactions

Examination of the temporal components showed that the relation of warm months and weekends with HFE appeared in Boston but not in Yokohama (Table 6). This may be explained in part by differences in the climatic conditions of the two locations and thus climate may matter.

The climate of Boston area is continental humid, with warm summers and winters that are cold, including snow. In Yokohama, the humid subtropical climate zone dominates, so that summers are hot and humid and winters are cool with occasional cold periods. Thus, spring (March–May) and autumn (September–November) are the most comfortable seasons in Japan. Spring is the time for *Hanami*, cherry-blossom viewing events, which are held in many different locations in Japan^54,55^. From the end of May to mid-July is the rainy season, which is called *Tsuyu*. During that period, even when it is not raining, the humidity increases as the temperature rises. When the rainy season ends, the pronounced hot weather begins. Unlike the challenges presented by the cold weather of Boston, the summer in Japan is indeed challenging because of the rainy days and the high humidity^56^.

Viewing cherry blossoms increases the happiness of subjects in some cities in Japan during the winter season which may affect the facial expressions in uploaded photos this season^54^. In addition, the suicide rate in Japan is higher during the summer months and lower during the winter^56,57^.

In Boston, the results show a clear negative relation between the snowy and cold winter with HFE. This may be because winters can be very harsh and demanding and therefore HFE prevalence may be reduced due to climatic discomfort.

The stronger relation between weekends and HFE in Boston may also be partly attributed to the difference in working hours between the two cultures. Although we could not find evidence for it in the scientific literature, Japan is widely known to have some of the longest working hours in the world. The Japanese legal system even recognizes the term “karoshi”—death by overwork—among the Japanese. In this working climate, Japanese people often take a low percentage of their paid vacation days and feel guilty when they actually take them. Therefore, the distinction between working days and weekends may be less pronounced in Yokohama compared with Boston, and consequently HFE prevalence more uniform among week days in the former.

### Presence of other individuals

The proportion of people with happy expressions was greater in the Boston photos than in Yokohama. This is consistent with previous research that found that Japanese individuals generally emotionally restrained^22,23,47^.

Another question was to what extent individuals from the two cultural contexts respond differently to ETN when they are photographed alone, as opposed to together with other people. The results show that the greater the number of people in the photo, the larger the proportion of happy faces, in both Boston and Yokohama. This means that in both societies, the tendency to smile is associated with the presence of more other people.

Surprisingly, a stronger association with number of people was found in Boston. This finding differs from previous research, which found that, compared to North Americans, Japanese individuals were more emotional or happier in the context of relationships^26^. That is, despite the fact that in collectivist societies happiness is likely to be experienced and expressed in the context of relationships between individuals.

One explanation of this interesting finding may be related to one’s relationship with the other people in the pictures (in-group versus out-group). Members of less collectivistic cultures have more in-groups, but they are less attached to any particular in-group^58,59^ and they tend to express more positive emotions (including happiness) to individuals from their in-group and from the out-group but this study cannot confirm this since we do not have enough information about the nature of the relationships between the people in the uploaded photos. Yet, more work is still needed to further explore the effect of others on the response of humans by happy facial expressions to exposure to nature.

### Implications, limitations, and future work

Our findings expand the scope of knowledge about the link between exposure to nature and happiness—as embodied in happy facial expressions—by showing that the relation holds in both more collectivist and less collectivistic societies. This work helps to illuminate the question as to whether the effects of cultural, educational or social activities are strong enough to mask individual expressions to exposure to nature. Additionally, these results were obtained on a very large sample, using a non-intrusive method.

The importance of ETN as a source of increased happiness is supported^6^, and politicians and the public have recently stressed the centrality of well-being and happiness to our lives, especially during an era when large parts of the population have more free time and the financial means to use it^60^. The present results suggest that despite cultural differences, exposure to nature acts as a powerful tool for increasing happiness in humans. Given that being in nature may increase happiness, both therapeutic treatments and government policies could be used to increase exposure to nature, especially in an era of greater buffers between humans and nature^61^. These initiatives can be applied different societies.

Two limitations of this study are that its evidence is insufficient to argue for causal conclusions, and the fact that our comparative data between nations cannot determine whether the effects are caused by the disparity between the two cultures or by other factors^20,62^.

Future research on the universality of the link should include replication efforts in similar pairs of societies, perhaps Germany and China, and comparable studies of other societies. For the latter, more detailed analysis of seasonal data would be needed than the dichotomous division of warm and cold months in the current research because in other parts of the world seasonal effects may have more subtle characteristics. Examples of this are desert societies and societies near the equator.

## Conclusions

Our data support the hypothesis of a universal effect of ETN on HFE, at least for the two culturally different cities studied. ETN-HFE relation appears to hold in both cities and the cities vary on several dimensions including the cultural aspect. This is the most important finding of this work and it expands the literature on the relation between exposure to nature and human emotion in the debate about universality versus culturally-based emotion. Specifically, the study suggests that green areas are more appreciated by the more collectivist Japanese society while proximity to water, weekends and hot months are more appreciated in the American society.

Another important finding is that in both societies, HFEs were more prevalent in the presence of others, but the relation was stronger in the American society. These results may inform governments and the general public about possible sites and timing to increase happiness and well-being by interacting with nature.

## Supplementary Information

Supplementary Information.

## Data Availability

The raw data used in all presented analyses can be downloaded from the following link—https://drive.google.com/file/d/1m1P_gt0SGSi_7ZQwj6n03AlZwvdLD-a7/view?usp=sharing. This is a Comma Separated Values (CSV) file. Each row represents a Flickr photo (n = 132,415 rows). The columns represent photo characteristics, as follows: area—Study area, either “Boston” or “Yokohama”, date—Date when photo was captured, x_utm—Photo X coordinate, in UTM, y_utm—Photo X coordinate, in UTM, ndvi—NDVI value, water—Distance from water (in meters), undeveloped—Whether location is developed (TRUE/FALSE), daytime—Whether photo was captured in daytime (TRUE/FALSE), weekend—Whether photo was captured during the weekend (TRUE/FALSE), warm—Weather photo was captured during the warm season (TRUE/FALSE), n_people—Number of people in the photo, emotion1—Emotion expressed in the photo ("neutral"/"happiness"), vegetation—Whether photo was captured in vegetated location (TRUE/FALSE), classified based on “ndvi”, water1—Whether photo was captured in proximity to water bodies (TRUE/FALSE), classified based on “water”, happy—Whether photo was expresses happiness (TRUE/FALSE), classified based on “emotion1”, composite—Composite ETN score, sum of “undeveloped”, “vegetation” and “water1”, area_date—Identifier of ”area” + “date”.
