# Supplementary material for: Nature and happiness in an individualist and a collectivist culture
Source: Sci Rep. 2022 May 11;12:7701. doi: 10.1038/s41598-022-11619-5 (PMC9095681; doi:10.1038/s41598-022-11619-5)
Supplement: Supplementary file 1 — Supplementary Information. [file 41598_2022_11619_MOESM1_ESM.docx]

**Nature and Happiness in an Individualist and a Collectivist Culture**

Tal Svoray^1, 2*^, Michael Dorman^1^, Sarah Abu-Kaf^3^, Golan Shahar^2^, Robert Gifford^4^

^1^ Department of Geography and Environmental Development, Ben-Gurion University of the Negev

^2^ Department of Psychology, Ben-Gurion University of the Negev

^3^ Conflict Management & Resolution Program, Ben-Gurion University of the Negev

^4^ Department of Psychology, University of Victoria

* Corresponding author

Tal Svoray, Ph.D.

Dept. of Geography and Environmental Development

Department of Psychology

Ben-Gurion University of the Negev, Beer-Sheva, ISRAEL

Phone # 972-8-6472018; Mobile # 972-525793052

Skype: tsvo10

http://www.geog.bgu.ac.il/gis/

tsvoray@bgu.ac.il

**Supplementary tables**

Table S1 – Confidence intervals for the fixed effect estimates listed in Tables 2-5.

| model | term | lower | est. | upper |
| --- | --- | --- | --- | --- |
| Composite | (Intercept) | -1.42 | -1.32 | -1.22 |
| Composite | areaYokohama | 0.7 | 0.82 | 0.94 |
| Composite | composite | 0.16 | 0.19 | 0.23 |
| Composite | n_people | 0.7 | 0.74 | 0.77 |
| Composite | daytime | -0.02 | 0.03 | 0.08 |
| Composite | weekend | 0 | 0.08 | 0.15 |
| Composite | warm | 0.09 | 0.16 | 0.24 |
| Composite | areaYokohama:composite | -0.07 | -0.03 | 0.01 |
| Composite | areaYokohama:n_people | -0.51 | -0.46 | -0.41 |
| Composite | areaYokohama:daytime | -0.07 | 0 | 0.07 |
| Composite | areaYokohama:weekend | -0.22 | -0.12 | -0.02 |
| Composite | areaYokohama:warm | -0.32 | -0.22 | -0.12 |
| Undeveloped | (Intercept) | -1.16 | -1.07 | -0.99 |
| Undeveloped | areaYokohama | 0.53 | 0.64 | 0.75 |
| Undeveloped | undeveloped | 0.24 | 0.29 | 0.34 |
| Undeveloped | n_people | 0.7 | 0.74 | 0.77 |
| Undeveloped | daytime | -0.02 | 0.03 | 0.08 |
| Undeveloped | weekend | 0 | 0.08 | 0.15 |
| Undeveloped | warm | 0.09 | 0.16 | 0.23 |
| Undeveloped | areaYokohama:undeveloped | 0.07 | 0.14 | 0.22 |
| Undeveloped | areaYokohama:n_people | -0.51 | -0.47 | -0.42 |
| Undeveloped | areaYokohama:daytime | -0.06 | 0.01 | 0.07 |
| Undeveloped | areaYokohama:weekend | -0.22 | -0.12 | -0.02 |
| Undeveloped | areaYokohama:warm | -0.32 | -0.22 | -0.12 |
| Water | (Intercept) | -1.22 | -1.13 | -1.04 |
| Water | areaYokohama | 0.56 | 0.68 | 0.79 |
| Water | water | 0.1 | 0.15 | 0.2 |
| Water | n_people | 0.69 | 0.73 | 0.77 |
| Water | daytime | -0.01 | 0.04 | 0.09 |
| Water | weekend | 0.02 | 0.09 | 0.17 |
| Water | warm | 0.11 | 0.18 | 0.26 |
| Water | areaYokohama:water | 0 | 0.07 | 0.14 |
| Water | areaYokohama:n_people | -0.51 | -0.46 | -0.41 |
| Water | areaYokohama:daytime | -0.05 | 0.02 | 0.09 |
| Water | areaYokohama:weekend | -0.23 | -0.13 | -0.03 |
| Water | areaYokohama:warm | -0.34 | -0.24 | -0.14 |
| Vegetation | (Intercept) | -1.16 | -1.07 | -0.98 |
| Vegetation | areaYokohama | 0.53 | 0.65 | 0.76 |
| Vegetation | vegetation | 0.02 | 0.07 | 0.12 |
| Vegetation | n_people | 0.69 | 0.73 | 0.77 |
| Vegetation | daytime | -0.02 | 0.03 | 0.08 |
| Vegetation | weekend | 0.01 | 0.09 | 0.16 |
| Vegetation | warm | 0.11 | 0.18 | 0.26 |
| Vegetation | areaYokohama:vegetation | -0.04 | 0.03 | 0.09 |
| Vegetation | areaYokohama:n_people | -0.51 | -0.46 | -0.42 |
| Vegetation | areaYokohama:daytime | -0.02 | 0.05 | 0.11 |
| Vegetation | areaYokohama:weekend | -0.23 | -0.13 | -0.03 |
| Vegetation | areaYokohama:warm | -0.34 | -0.24 | -0.15 |

**Supplementary figures**

Figure S1 – Proportion of images with happy expression per study area per level of examined environmental variable and number of people in the photo. The labels above each bar specify the proportion (out of images with happy+neutral expression) and the count of images with happy expression.
